# Gastrointestinal mucormycosis by *Mucor indicus*: A report of two cases

**DOI:** 10.1016/j.mmcr.2025.100693

**Published:** 2025-01-20

**Authors:** Alex Rivero, Megan Shaughnessy, Jessica Oswald, Nicholas Goodhope, Margret Oethinger

**Affiliations:** a Division of Infectious Disease/Department of Medicine, Hennepin County Medical Center, 701 Park Ave S, Minneapolis, MN, 55415, United States; b Microbiology and Molecular Diagnostics, Department of and Laboratory Medicine and Pathology, Hennepin County Medical Center, 701 Park Ave S, Minneapolis, MN, 55415, United States

**Keywords:** *Mucor indicus*, Mucormycosis, Gastrointestinal tract

## Abstract

Mucormycosis is an invasive infection caused by fungi of the order Mucorales, typically affecting immunocompromised individuals, and rarely involving the gastrointestinal tract. We report two cases of gastrointestinal mucormycosis by *Mucor indicus:* a 77-year-old woman with a gastric ulcer and a 25-year-old man with liver lesions. Both were treated with surgery and liposomal amphotericin B; only one survived. Recognizing gastrointestinal mucormycosis in the correct clinical context is essential and requires timely surgical and antifungal treatment.

2012 Elsevier Ltd. All rights reserved.

## 1 Introduction

Fungi of the order Mucorales are saprophytic fungi that can cause severe infections in immunocompromised patients. The members of the family Mucoraceae isolated most commonly are *Rhizopus* spp., followed by *Mucor* as the second most common genus [1]. In contrast to other filamentous, opportunistic fungi that infect immunosuppressed patients, such as patients with cancer or organ transplants, Mucorales also infect patients with milder immunodeficiencies such as diabetes mellitus, people who inject drugs, patients on deferoxamine therapy, or those with no known underlying conditions [1]. The infection pathway is airborne, skin inoculation, or ingestion of sporangiospores.

We observed two patients at our hospital infected with an unusual species of Mucorales, *Mucor indicus*, in their gastrointestinal system. Infection was diagnosed either histologically or by culture and confirmed with molecular methods.

## 2 Case presentations

### 2.1 Case 1

**Fig. 1:**
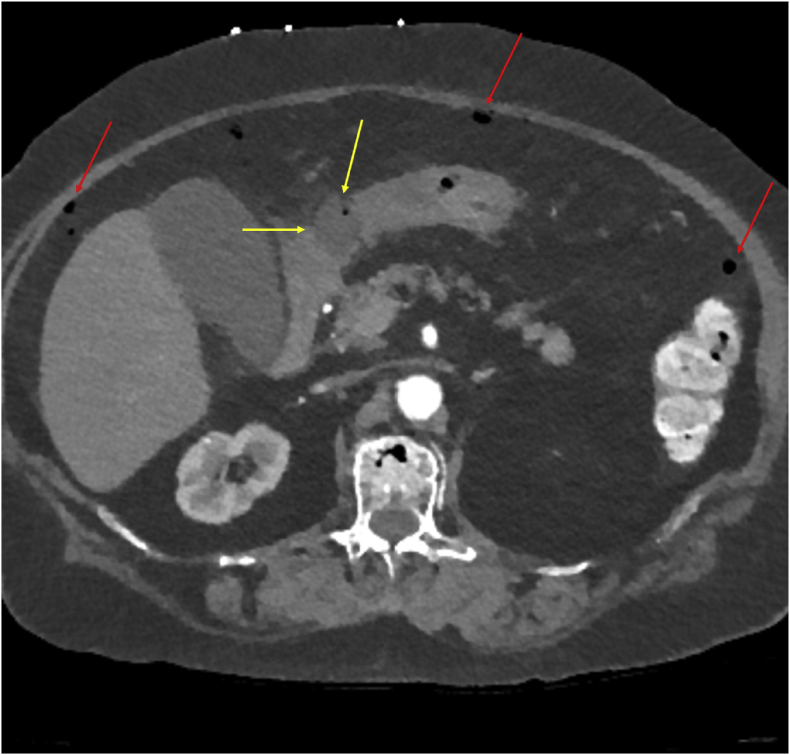
Yellow arrows delineate the focal discontinuity suggestive of a perforated gastric ulcer, while the red arrows highlight areas of free air.

A 77-year-old woman originally from Laos with asthma, type 2 diabetes mellitus, and peptic ulcer disease presented on Day 0 in with a 1-week history of upper abdominal and chest pain, nausea, constipation, generalized weakness, and decreased appetite. Vitals were notable for hypotension (90/64 mmHg) and lack of fever. The physical exam demonstrated right upper quadrant tenderness. Laboratory evaluation showed a drop in her hemoglobin to 7.5 g/dL from 11.4 g/dL measured on day −5. Abdominal CT scan with IV contrast revealed pneumoperitoneum and intraperitoneal free fluid. There was also a focal discontinuity in the wall of the gastric antrum, which was suspected to represent a perforated gastric ulcer (Fig. 1).

The patient was taken urgently to the OR for exploratory laparotomy on Day 0 and ultimately had an antrectomy, selective vagotomy, open cholecystectomy, loop gastrojejunostomy, and abdominal drain placement. Intraoperative findings were remarkable for a 4 cm in diameter defect in the anterior gastric wall. She was started on piperacillin/tazobactam postoperatively and admitted to the surgical intensive care unit due to hypotension requiring vasopressors. She was extubated on Day +3.

**Fig. 2:**
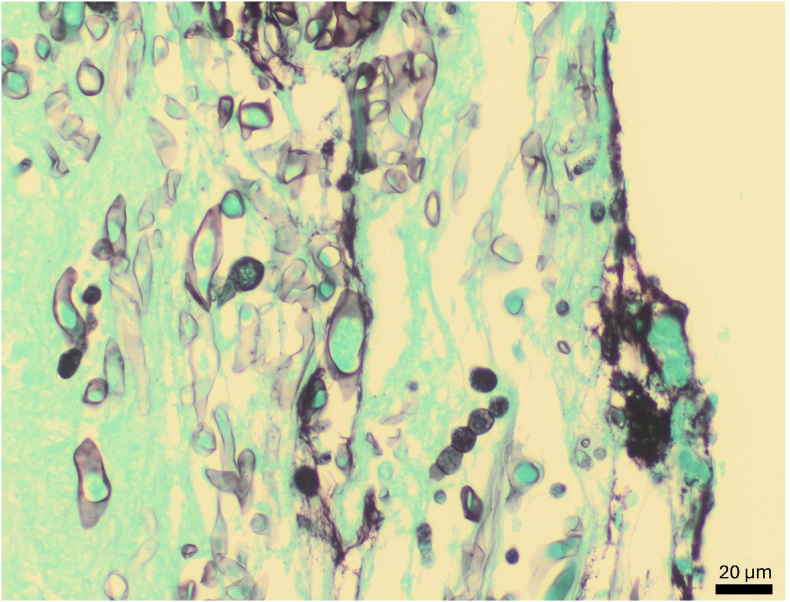
Pauciseptate fungal hyphae with irregularly shaped walls and 90-degree branching. Intercalary chlamydoconidia, both singly and in chains, were present, measuring 10–15 μm in diameter.

Surgical pathology revealed an acute transmural ulceration and numerous invasive hyaline hyphae within the gastric wall of the antrum on H&E stain. Morphology on GMS stain was consistent with a fungus of the family *Mucoraceae* (see Fig. 2). There was no fungal culture obtained at the time of surgery to correlate with the histopathologic findings.

The patient's postoperative course was complicated by worsening respiratory status, requiring re-intubation. The infectious disease consultant was involved on Day +9, once histopathology results returned, and she was started on liposomal amphotericin B at 10 mg/kg/IV daily. However, given worsening renal dysfunction, she was switched to isavuconazole 372 mg IV every 8 hours for six doses and then 372 mg IV daily on Day +13. She was extubated once again on Day +15. Fungal identification as *Mucor indicus* was obtained by broad-range PCR and Sanger sequencing on the formaldehyde-fixed paraffin-embedded (FFPE) tissue block at the University of Washington, Seattle, Washington. Targets were the ribosomal subunit 28S and the internal transcribed spacers ITS1 and ITS2 [2]. The least sensitive target, 28S, did not detect any template but sequences of *Mucor indicus* were detected by targeting ITS1 and ITS2. After an approximately two-month hospital admission, the patient was discharged with plans to continue isavuconazole for invasive mucormycosis for a total antifungal course of 3 months from surgery. Surgery was deemed successful at achieving source control, given viable margins were seen on tissue pathology.

Three months after being discharged from the hospital, she was admitted to an outside hospital and found to have recurrent gastric perforation. The patient refused to undergo surgery but had an upper endoscopy, which revealed diffuse ulceration with eschar and white plaques at the GJ anastomosis. Additionally, multiple white plaques were seen extending proximally into the stomach. Multiple biopsies were taken from the jejunum, GJ anastomosis, and stomach. Jejunal biopsy returned positive for fungal organisms, though identification was impossible by histopathology alone. Given the clinical history, there was concern for recurrent invasive mucormycosis, for which the patient was placed on IV liposomal amphotericin B. The infectious disease consultant was involved, and surgery was recommended as the only means to achieve source control. The patient declined surgery and wished to be transitioned to comfort care; she died two weeks after discharge.

The etiology of exposure to *Mucor* was unknown. The final diagnosis was that of gastric ulcer with perforation secondary to invasive mucormycosis by *Mucor indicus*.

### 2.2 Case 2

A 25-year-old man originally from Thailand with end-stage renal disease secondary to IgA nephropathy underwent a deceased donor kidney transplantation. He received a high-risk induction immunosuppression protocol with four days of anti-thymocyte globulin along with initiating steroids, tacrolimus, and mycophenolate mofetil. He commenced prophylactic antimicrobials, including valganciclovir (CMV donor and recipient positive) and trimethoprim-sulfamethoxazole. His immediate postoperative course was complicated by delayed graft function and hyperkalemia requiring hemodialysis.

**Fig. 3:**
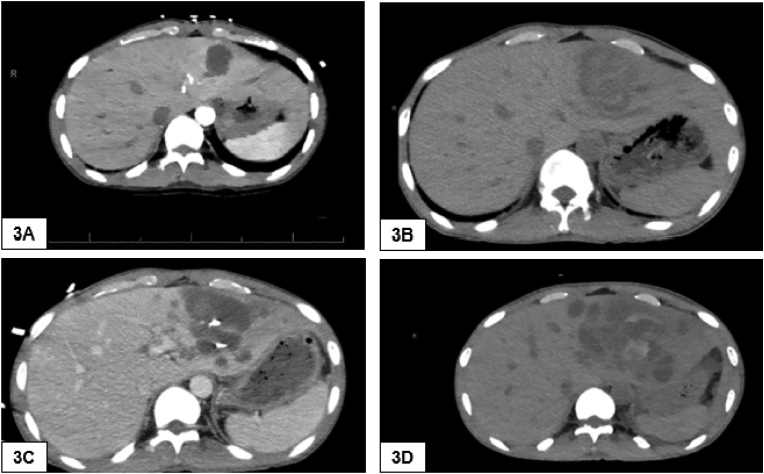
**(3A)**. 2.9 cm fluid density in the left lobe of the liver **(3B).** Complex fluid collection in the left lobe of the liver with surrounding hypodense satellite lesions, significantly increased from prior. **(3C).** Interval increase in the complex fluid collection without evidence of extravasation; satellite lesions also increased in size and number. **(3D).** Increase in satellite lesions around primary stable fluid collection.

On Day +24, he presented to the transplant clinic with approximately one day of right-sided lower pleuritic-type chest pain, generalized abdominal pain, and dyspnea. A CT pulmonary angiogram was performed that excluded a pulmonary embolism, but he was incidentally found to have a new 2.9 cm fluid collection in the left lobe of the liver, suspected to be a liver cyst (Fig. 3A). Given the transient improvement in his symptoms, he was discharged home.

On day +31, he returned to the transplant clinic with an exacerbation of the previous symptoms; his abdominal pain was now localized to the right upper quadrant, and he was febrile (38.8 °C). Laboratory values were notable for mild leukocytosis (12.53 m/cm). CT of the abdomen was obtained, and a complex fluid collection that was significantly increased in size compared to the CT from day +24 was seen (see Fig. 3B).

He was hospitalized and underwent immediate placement of a drain by interventional radiology, which returned with frank red blood. A follow-up triple-phase CT scan of the abdomen obtained on the same day showed active extravasation of blood, for which he underwent coiling and embolization of multiple branches of the left lobe of the liver. Aspirate was sent for aerobic and anaerobic bacterial, fungal, and acid-fast bacillus cultures. He was started on piperacillin/tazobactam. Despite antibiotics, the patient remained persistently febrile. Blood cultures and cultures from the initial liver fluid aspirate remained without growth. 16S rDNA sequencing of the aspirate failed to detect a bacterium. Repeat abdominal CT imaging on Day +35 was obtained and showed an interval increase in the complex fluid collection and the satellite lesions (Fig. 3C).

Piperacillin/tazobactam was discontinued on Day +38, given no apparent benefit to the clinical course in the setting of negative bacterial cultures. A broad-range PCR for fungi from the initial aspirate was sent to a reference laboratory at the University of Washington.

**Fig. 4:**
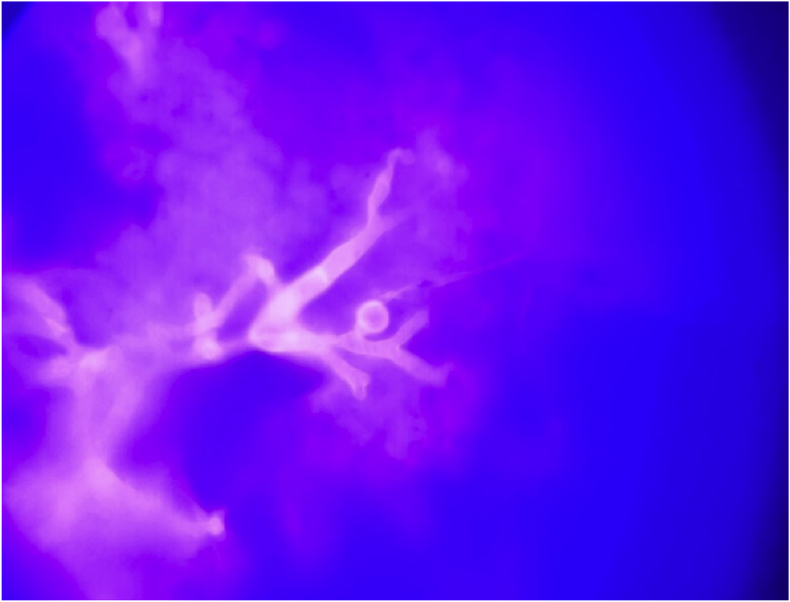
Fungal stain of the direct specimen showing broad, distorted hyphae.

On Day +40, a repeat culture obtained from the indwelling drain showed fungal elements on a direct fungal stain. A filamentous fungus grew on Day +41, phenotypically identified to the genus level as *Mucor* spp. (Fig. 4). Subsequently, broad-range PCR and 28S, ITS1 and ITS2 Sanger sequencing at the University of Washington laboratory identified sequences of *Mucor indicus.*

The fungal isolate was sent for species level identification and antimicrobial susceptibility testing to ARUP Laboratory, Salt Lake City, Utah. Identification by Matrix-Assisted Laser Desorption Ionization – Time of Flight (MALDI-TOF) failed. The isolate was confirmed as *Mucor indicus* by ITS Sanger sequencing (primers FITS5-M13T and FITS4R-M13T). The sequences were not submitted by either University of Washington nor ARUP to the GenBank or any other public database. On Day +41 Mycophenolate mofetil was discontinued, and empiric liposomal amphotericin B was started at 10 mg/kg. CT of the head, sinuses, and chest showed no abnormalities to suggest disseminated mucormycosis. Despite antifungal treatment, repeat abdomen imaging on Day +45 showed increased sizes of the satellite lesions (Fig. 3D).

**Fig. 5:**
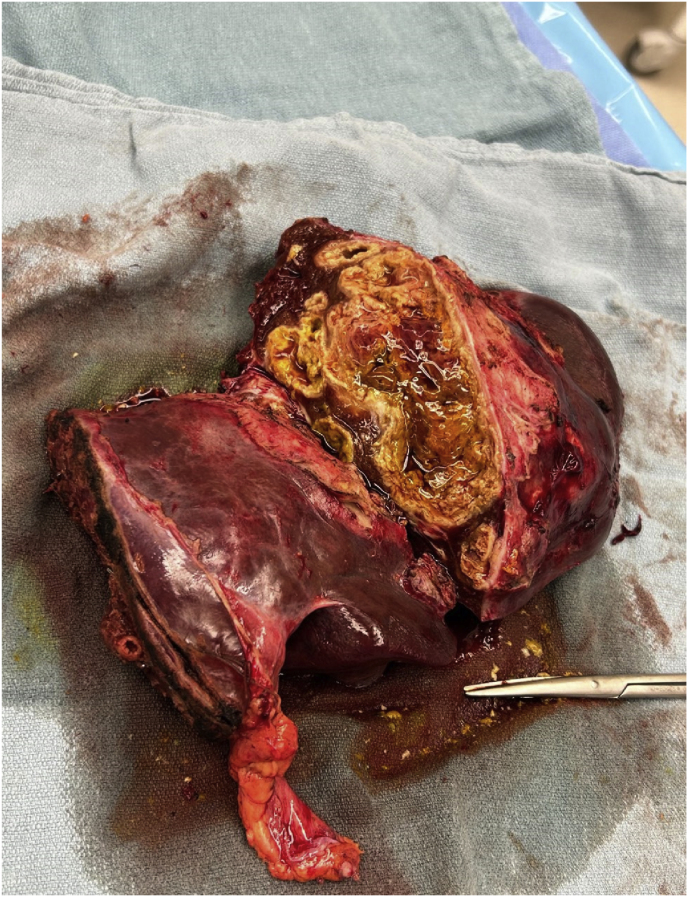
The gross appearance of the left hepatectomy revealed an 8.0 × 5.0 cm of adherent, shaggy fibrous tissue, which was tan-gray and partially softened. The specimen was sectioned to reveal a multifocal tan-yellow, poorly circumscribed, centrally cavitated necrotic abscess.

Given concerns for lack of source control, and per guidelines for management of mucormycosis, surgical resection was pursued. On Day +59 the patient underwent a right liver hepatic lesion wedge resection, left hepatectomy, and cholecystectomy (Fig. 5).

**Fig. 6:**
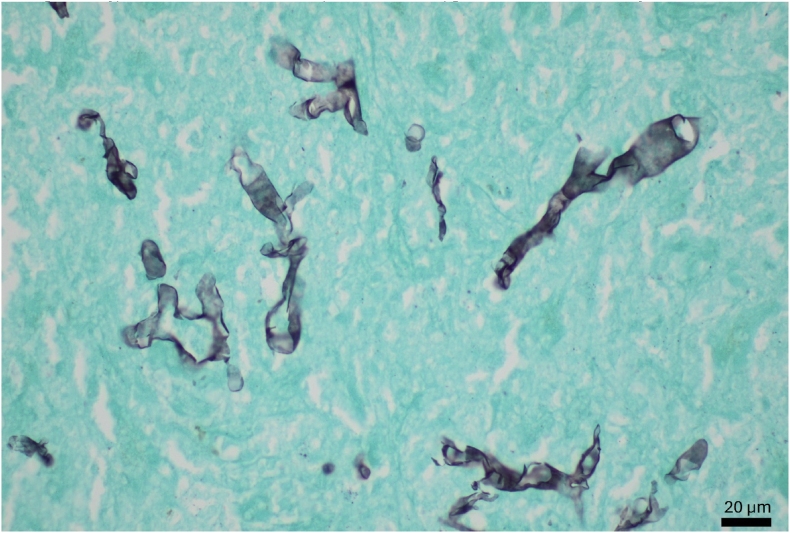
GMS stain reveals pauciseptate, broad, irregular hyphae consistent with a member of the family *Mucoraceae.*

Histopathology of liver tissue showed hyaline, broad hyphae on H&E stain (Fig. 6).

Susceptibility was performed by broth microdilution, with the following results: Fluconazole and voriconazole: intrinsically resistant; MIC (μg/mL) 1 for liposomal amphotericin B and ≥16 for isavuconazole, itraconazole and posaconazole, respectively. No interpretation guidelines of the Clinical and Laboratory Standards Institute for interpretation of these MIC-species combinations exist. The patient was discharged home on Day +77 to continue liposomal amphotericin B infusions. Abdominal imaging obtained on Day +118 showed interval resolution of the mixed fluid collection with stable postsurgical changes and no new findings to suggest spreading infection. He completed three months of liposomal amphotericin B post-liver resection (and four months of antifungals total). However, given the development of nephrotoxicity attributed to the liposomal amphotericin B, the decision was made to discontinue his antifungal treatment. The patient has not shown signs of recurrent infection to date.

The exact exposure to *Mucor indicus* in this patient is unclear. It is possibly linked to the ingestion of naturopathic medication and tea, which he ordered from Thailand. The final diagnosis was that of isolated hepatic mucormycosis from *Mucor Indicus*.

## 3 Discussion

Amongst *Mucor* spp., *Mucor indicus* has infrequently been described; whether this is due to the lack of *Mucor* species identification or lack of recovery from clinical specimens is unknown [1,3]. Clinical laboratories frequently report members of the family of Mucoraceae at the genus level (*Rhizopus* spp., *Rhizomucor* spp., *Mucor* spp.) or even as a 'member of the order Mucorales'. Neither genus nor species identification is possible by histopathology. For this reason, the actual spectrum of species of mycomycetes and their relative frequencies of isolation from patients is not well known. To obtain this knowledge, a large mycological reference center (Fungus Testing Laboratory, University of Texas Health Science Center), which receives fungal isolates from all over the United States of America, performed a study on 190 consecutively received clinical isolates [4]. The most common species was *Rhizopus oryzae*, comprising nearly half of the isolates tested (44.7 %), followed by *Rhizopus microspores* (22.1 %), *Mucor circinelloides* (9.5 %), *Mycocladus corymbifer* (5.3 %), *Rhizomucor pusillus* (3.7 %), *Cunninghamella bertholletiae* (3.2 %), *Mucor indicus* (2.6 %), *Cunninghamella echinulata* (1 %), and *Apophysomyces elegans* (0.5 %) [4]. These results align with the observation that another reference laboratory identified *Mucor indicus* only four times within the past 2.5 years [personal communication], indicating that *Mucor indicus* is indeed a rarely isolated fungus in clinical laboratories.

Gastrointestinal manifestations of mucormycosis account for only 7 % of all cases [1], with the stomach being the most frequently affected organ [3].

The two invasive *Mucor indicus* infection cases presented here involved gastrointestinal tract organs, specifically the stomach and liver, and occurred within four months of each other at our institution but were epidemiologically unrelated. Similar cases to ours have been described in the literature. A case study in 2005 described eight cases of *Mucor indicus*, of which five patients presented with gastrointestinal involvement, including a case of an epigastric mass found to be a gastric ulcer, two cases with multiple irregular hepatic abscesses, a case with cecal wall thickening, and a case with gastric and intestinal necrosis and ulceration [5]. The three remaining cases had unique infection locations: vaginitis, necrotizing fasciitis of the left knee, and an implantable left ventricular assist device infection [5]. In 2019, Uchida T et al. reported a case of gastric mucormycosis by *Mucor indicus,* which developed secondary to immunosuppressive treatment for adult-onset Still's disease [6]. Although the patient was treated with antifungal drugs, the gastric ulcer developed into a gastropleural fistula, and the patient died due to pneumonia [6]. Uchida T. et al. also reviewed 19 cases of gastric mucormycosis (*Rhizopus* spp. in 6 cases, *Mucor indicus* in 1 case, and unidentified fungi in 12 cases), of which nine cases were fatal (47 %) [6]. Lastly, a case of disseminated mucormycosis including the liver caused by *Mucor indicus* was reported in a patient with AML undergoing chemotherapy [7]. Cultures were negative, and *Mucor indicus* was identified by metagenomic next-generation sequencing from the patient's blood [7].

The frequent involvement of the gastrointestinal tract raises the question of whether oral ingestion of fungal spores was the portal of entry in our and other cases. Interestingly, *Mucor indicus* is used in the food industry: *Mucor indicus* was one of several members of the Mucoraceae that were isolated from peka, which is used as a commercial wine starter and as fermented food in Taiwan, with an alcoholic sweet taste and a fruit flavor [8]. In the case report by Oliver M et al., a bone marrow transplant recipient with graft-versus-host disease treated with prednisone developed *Mucor indicus* liver abscesses after ingestion of naturopathic medicine [9]. *Mucor indicus* was isolated – among other bacteria and molds – from the patient's residual naturopathic medicine, and randomly primed PCR analysis showed that the medicinal and patient isolates were clonally related [9]. Only one of our two cases had a potential connection with food. The case 2 patient reported regularly ingesting a drink from leaves obtained from Thailand, however no product was available for testing. We hypothesize that the point of entry of the infectious agent was via oral ingestion of fungal sporangiospores, potentially related to the ingestion of imported naturopathic medicine or food products.

Current management guidelines for mucormycosis indicate the need for aggressive therapy combining surgical debridement and antifungal treatment. Liposomal amphotericin B remains the first-line antifungal, with doses of 5–10 mg/kg/day strongly recommended. The optimal duration of therapy is uncertain but often requires weeks to months of treatment [10].

In summary, our cases show a rare manifestation of an infection by an uncommon opportunistic fungus. Considering gastrointestinal mucormycosis in the correct clinical context is essential, as timely surgical intervention and antifungal therapy are critical in improving outcomes.

## CRediT authorship contribution statement

**Alex Rivero:** Writing – review & editing, Writing – original draft. **Megan Shaughnessy:** Writing – review & editing. **Jessica Oswald:** Visualization. **Nicholas Goodhope:** Visualization. **Margret Oethinger:** Writing – review & editing, Writing – original draft.

## Funding

This research did not receive any specific grant from funding agencies in the public, commercial, or not-for-profit sectors.

## Declaration of competing interest

None of the authors report a conflict of interest.
